# Outcomes of Platinum plus /Pemetrexed Rechallenge in Pleural Mesothelioma: The Role of Platinum-Free Interval in a Multicenter Real-World Analysis

**DOI:** 10.1016/j.jtocrr.2026.101073

**Published:** 2026-08-31

**Authors:** Rajiv Shah, Martin Reck, Christian Grohé, Martin Faehling, Daniel C. Christoph, Felix C. Saalfeld, Eva L. Buchmeier, Hans-Georg Kopp, Petra Hoffknecht, Nikolaj Frost, Frank Griesinger, Jonas Kuon, Jingting Luan, Julia Roeper, Martin Kimmich, Till Olchers, Paolo Chesi, Laura V. Klotz, Inn Chung, Helge Bischoff, Michael Thomas, Petros Christopoulos

**Affiliations:** a Department of Thoracic Oncology, Thoraxklinik, Heidelberg University Hospital and National Center for Tumor Diseases (NCT), NCT Heidelberg, a partnership between DKFZ and Heidelberg University Hospital, Germany; Translational Lung Research Center Heidelber, Member of the German Center for Lung Research, Heidelberg, Germany; b Lung Clinic Grosshansdorf, Airway Research Center North, German Center for Lung Research (DZL), Grosshansdorf, Germany; c Klinik für Pneumologie -Evangelische Lungenklinik Berlin Buch, Berlin, Germany; d Department of Pneumology, Klinikum Esslingen GmbH, Esslingen, Germany; e Thoraxzentrum Ruhrgebiet, Klinik für Pneumologie und Infektiologie, Sektion Thoraxonkologie, Ev. Krankenhaus Herne, Akad. Lehrkrankrankenhaus Ruhr-Universität Bochum, Herne, Germany; f Department of Medicine I, University Hospital Carl Gustav Carus and NCT/UCC Dresden, a partnership between DKFZ, Faculty of Medicine and University Hospital Carl Gustav Carus, TUD Dresden University of Technology, and Helmholtz-Zentrum Dresden-Rossendorf, Germany; g Klinikum der Stadt Köln gGmbH, Cologne, Germany; h Robert Bosch Centrum für Tumorerkrankungen, Klinik Schillerhöhe, Stuttgart, Germany; i Franziskus Hospital Harderberg, Niels-Stensen-Kliniken, Department of Thoracic Oncology, Georgsmarienhütte, Germany; j Charité–Universitätsmedizin Berlin, Corporate Member of Freie Universität Berlin, Humboldt-Universität zu Berlin, and Berlin Institute of Health, Berlin, Germany; k Department of Hematology and Oncology, University Department Internal Medicine-Oncology, Pius-Hospital, University Medicine Oldenburg, Oldenburg, Germany; PIUS Hospital Oldenburg, Universitätsklinik für Innere Medizin-Onkologie, Oldenburg, Germany; l SLK Fachklinik Löwenstein, Löwenstein, Germany; m Department of Oncology and Dermatological Oncology, IDI-IRCCS, Rome, Italy; n Department of Thoracic Surgery, Thoraxklinik, Heidelberg University Hospital and NCT, NCT Heidelberg, a partnership between DKFZ and Heidelberg University Hospital, Germany; Translational Lung Research Center Heidelberg, Member of the DZL, Heidelberg, Germany

**Keywords:** Chemotherapy, Immunotherapy, Mesothelioma, Pemetrexed, Platinum-free interval, Rechallenge

## Abstract

**Introduction:**

Real-world (rw) practice of rechallenge with platinum plus pemetrexed (PP) in pleural mesothelioma (PM) has been recommended in international guidelines, but the evidence is scarce and based on a few very small retrospective studies.

**Methods:**

In total, 612 patients with PM treated with first-line PP between 2010 and 2023 across 12 centers were retrospectively identified. Outcomes and treatment patterns were analyzed across second-line regimens, with a particular focus on PP rechallenge. The platinum-free interval (PFI) was evaluated using a two-tier classification (platinum-sensitive ≥6 mo and platinum-resistant <6 mo) as a selection criterion.

**Results:**

Among 375 patients (61%) receiving second-line therapy, the rw median overall survival (rw-mOS) and rw median progression-free survival (rw-mPFS) were 11.6 months and 4.7 months, respectively. Patients with PP rechallenge (n = 78) had a median OS of 20.5 months, a median rwPFS of 6.8 months, and a disease control rate of 88%. Patients with platinum sensitivity derived greater benefit from rechallenge, with rw-mOS differing across PFI categories (platinum-sensitive: 21.4 mo versus platinum-resistant: 11.9 mo; *p =* 0.007). Outcomes with nivolumab plus ipilimumab (n = 29) were comparable to those observed with PP rechallenge (rw-mOS = 22.8 mo, rw-mPFS = 8.0 mo), with treatment-free interval (TFI) associated with improved median OS in the platinum-sensitive group compared with the platinum-resistant group (32 mo versus 18.1 mo, *p =* 0.03). Single-agent pemetrexed (n = 32) and single-agent chemotherapy were associated with substantially shorter rw-mOS of 11.6 and 8.0 months, respectively.

**Conclusion:**

In conclusion, PP rechallenge has favorable outcomes and remains a viable alternative in patients with platinum sensitivity with PM, whereas nivolumab plus ipilimumab may be considered in both TFI categories, particularly in platinum-resistant disease, in which PP rechallenge offers limited benefit. A PFI or TFI-guided approach to second-line treatment selection is supported, although prospective validation is warranted.

## Introduction

Pleural mesothelioma (PM) is an aggressive malignancy arising from the pleural lining, with a poor prognosis, historically associated with asbestos exposure.^1^ The landmark phase III trial by Vogelzang et al.^2^ established cisplatin plus pemetrexed as the standard first-line regimen in PM, reporting a median overall survival (OS) of 12.1 months compared with 9.3 months with cisplatin alone. Recent phase III data using cisplatin or carboplatin in combination with pemetrexed in the control arm have reported a median OS of up to 18 months in patients with epithelioid histology.^3^^,^^4^ The addition of bevacizumab to cisplatin and pemetrexed resulted in an OS benefit in the phase III MAPS trial, although this combination has not received Food and Drug Administration or European Medicines Agency approval.^5^ The CheckMate-743 trial established nivolumab plus ipilimumab as a first-line option, with a distinct benefit observed in nonepithelioid histology. Notably, among patients with the epithelioid subtype, outcomes with platinum plus pemetrexed (PP) were comparable to those achieved with dual immune checkpoint inhibition.^4^ Furthermore, the addition of pembrolizumab to chemotherapy did not improve survival in the epithelioid subtype in the phase III IND.227 (KEYNOTE-483) trial.^3^ Importantly, real-world outcomes with nivolumab plus ipilimumab have been reported to be inferior to those observed in CheckMate-743, even among trial-eligible patients, and immune-related adverse events frequently necessitate early treatment discontinuation.^6^, ^7^, ^8^ Thus, although nivolumab plus ipilimumab represents the preferred option in the nonepithelioid subtype, PP has comparable efficacy in the epithelioid subtype and may be considered particularly in patients with the epithelioid subtype in which immune-related toxicity is a concern.

Beyond first-line therapy, no systemic regimen has received regulatory approval. Current guidelines recommend considering rechallenge with pemetrexed-based chemotherapy or other available active agents, including vinorelbine and gemcitabine. Treatment selection is often guided by prior therapy, histology, and performance status.^9^^,^^10^ Gemcitabine in combination with ramucirumab reports promising results in the randomized phase II RAMES trial, although this regimen remains investigational.^11^ In this context, off-label use of various agents is common in clinical practice.

Notably, various mechanisms of sensitivity and resistance to PP have been previously reported in PM and NSCLC.^12^, ^13^, ^14^, ^15^, ^16^, ^17^ Nevertheless, to our knowledge, platinum sensitivity has not been evaluated as a treatment-guiding factor in PM. Recent studies have characterized the genomic landscape of PM, revealing recurrent alterations in tumor suppressor genes, including *BAP1, CDKN2A,* and *NF2*.^18^^,^^19^ Moreover, *BAP1* loss has been reported as a predictive biomarker for response to PP and as a prognostic marker in PM.^20^ Nevertheless, the molecular profile is not yet incorporated into treatment algorithms.

Given the lack of approved second-line therapies, some small retrospective cohorts of fewer than 30 patients have explored rechallenge with pemetrexed-based chemotherapy (alone or in combination with a platinum compound).^21^, ^22^, ^23^, ^24^, ^25^ These analyses suggest that rechallenge may be feasible and potentially effective in a subset of patients. Nevertheless, the evidence remains limited to small retrospective series, with no randomized trial having addressed this question so far.

In this study, we investigated treatment patterns and outcomes in a real-world cohort of patients with PM treated with first-line PP chemotherapy, with a particular focus on the subgroup of patients who received PP rechallenge in the second-line setting and its association with the platinum-free interval (PFI).

## Materials and Methods

### Participants

This retrospective, multicenter study included patients from the German MesoNet Cohort, which comprises 1575 consecutive patients with PM from 12 participating German thoracic oncology centers. All patients were diagnosed between January 1, 2010, and December 31, 2023, and the data cutoff was on July 4, 2024. For this analysis, patients with a histologically confirmed diagnosis of PM were included if they received first-line PP chemotherapy followed by at least one subsequent line of systemic therapy. Patients who did not receive second-line therapy were included only in a descriptive analysis of treatment patterns and attrition. For eligible patients, demographic information, clinicopathologic features, baseline laboratory values, and outcome variables were extracted from medical records. Tumor stage was classified according to the eighth edition of the Union for International Cancer Control TNM staging system.

### Study Objectives

The primary objective of this study was to evaluate the efficacy of PP rechallenge in the second-line setting among patients with PM, assess its association with PFI, and describe treatment patterns and outcomes across all second-line regimens used. Real-world median OS (rw-mOS), real-world median progression-free survival (rw-mPFS), and, for patients for whom data were available, objective response rate were analyzed as primary efficacy end points. Treatment response was assessed according to the local standard per the modified Response Evaluation Criteria in Solid Tumors criteria for PM.

### Statistical Analyses

Continuous variables are presented as medians with interquartile ranges (IQRs). Age is presented as a mean with SD. Categorical variables are reported as counts and percentages. In the descriptive analysis, the proportion of each categorical parameter was calculated relative to the total number of nonmissing observations, whereas the proportion of missing values is reported relative to the total number of observations, including missing values. The start date of second-line treatment was assigned as the baseline date for all outcome analyses and for the calculation of median follow-up time (unless mentioned otherwise). Median follow-up time was estimated using the reverse Kaplan-Meier method. All outcomes were estimated using the standard Kaplan-Meier method. In this study, OS was defined as the time in months from the initiation of second-line treatment to death from any cause or to the date of previous contact for patients who were still alive at the time of analysis. rwPFS was defined as the time in months from the initiation of second-line treatment to documented disease progression or death from any cause, whichever occurred first.

In addition, PFI and treatment-free interval (TFI) were defined as the interval between the last administration of a platinum compound in first-line therapy and the initiation of second-line treatment. PFI was explicitly used for PP rechallenge, whereas TFI was used to denote the same interval for other second-line regimens. Given the exploratory and hypothesis-generating nature of this retrospective analysis, PFI and TFI were evaluated using a clinically informed two-tier classification—platinum-sensitive (≥6 mo) and platinum-resistant (<6 mo).

Survival curves and rates were estimated using the Kaplan-Meier method. Comparisons between groups were performed using the log-rank test, with statistical significance defined as *p <* 0.05. Hazard ratios (HRs) with 95% confidence intervals (CIs) were estimated using Cox proportional hazards regression. All analyses were performed on available data using a complete case approach. Subgroup analyses were exploratory and were not adjusted for multiple testing. Both univariable and multivariable Cox regression models were applied to identify covariates associated with survival outcomes. For continuous variables, the median was used as the cutoff value. For subgroups with insufficient events precluding Cox model convergence, HRs are reported as not estimable.

To evaluate the prognostic impact of blood-based immune-inflammatory markers, baseline cell ratios at the time of diagnosis were assessed. Neutrophil-to-lymphocyte ratio (NLR) was calculated as neutrophil count divided by lymphocyte count, and systemic immune-inflammation index was calculated as the product of platelet count multiplied by neutrophil count divided by lymphocyte count; both were stratified using thresholds previously established in PM.^26^ Platelet-to-lymphocyte ratio was calculated as platelet count divided by lymphocyte count, with patients stratified using the median value (236) derived from the overall MesoNet cohort (N = 1550) because no validated PM-specific threshold was available. The lung immune prognostic index was derived from a combination of derived NLR (dNLR = neutrophil count/[white blood cell count − neutrophil count]) and serum lactate dehydrogenase (LDH), stratifying patients into good (dNLR ≤ 3 and LDH ≤ upper limit of normal), intermediate (dNLR > 3 or LDH > upper limit of normal), and poor (dNLR > 3 and LDH > upper limit of normal) prognostic groups, as previously described.^27^

All statistical analyses were performed and figures generated using R version 4.5.2 (R Foundation for Statistical Computing, Vienna, Austria). This study was approved by the Ethics Committee of Heidelberg University (approval number: S-062/2019). Informed consent was waived because of the retrospective nature of the study.

## Results

### Patient Characteristics

Among the 1550 patients in the German MesoNet Cohort, 612 received first-line PP chemotherapy, of whom 78 were subsequently re-treated with PP in the second-line setting. Baseline characteristics for all patients treated with first-line PP are summarized in Supplementary Table 1. Among patients receiving second-line PP, 76.9% were men, and the mean age was 68 years, with 73% aged 65 years or older. Seventy patients (89%) had epithelioid histology, seven had biphasic histology, and one was classified as having other histology. Sarcomatoid histology was not represented in this subcohort. Prior asbestos exposure was documented in 78% of patients with available data; asbestos exposure status was not documented in 24% of the PP rechallenge cohort. Eastern Cooperative Oncology Group performance status (ECOG PS) was 1 or lower in all patients with available data (missing in 24%). Most of the patients had early-stage disease, and TNM stage was missing in 26% (Table 1).

**Table 1:** Patient Characteristics of the Platinum/Pemetrexed Rechallenge Subcohort

Parameter	Overall, N = 78^a^	Epithelioid, N = 70^a^	Non-Epithelioid, N = 8^a^
Sex			
Male	60 (76.9%)	55 (78.6%)	5 (62.5%)
Female	18 (23.1%)	15 (21.4%)	3 (37.5%)
Age (Mean, y)	68 (8)	68 (8)	63 (6)
Age			
< 65 y	21 (26.9%)	17 (24.3%)	4 (50.0%)
≥65 to <75 y	40 (51.3%)	36 (51.4%)	4 (50.0%)
≥75 y	17 (21.8%)	17 (24.3%)	0 (0.0%)
Histology			
Epithelioid	70 (89.7%)	70 (100.0%)	0 (0.0%)
Biphasic	7 (9.0%)	0 (0.0%)	7 (87.5%)
Sarcomatoid	0 (0.0%)	0 (0.0%)	0 (0.0%)
Other	1 (1.3%)	0 (0.0%)	1 (12.5%)
Smoking history			
Never	14 (28.6%)	12 (27.3%)	2 (40.0%)
Current	10 (20.4%)	9 (20.5%)	1 (20.0%)
Former	25 (51.0%)	23 (52.3%)	2 (40.0%)
Missing	29 (37%)	26 (37%)	3 (38%)
Asbestos exposure	46 (78.0%)	43 (81.1%)	3 (50.0%)
Missing	19 (24%)	17 (24%)	2 (25%)
ECOG PS Score			
0	25 (42.4%)	20 (37.7%)	5 (83.3%)
1	34 (57.6%)	33 (62.3%)	1 (16.7%)
≥ 2	0 (0.0%)	0 (0.0%)	0 (0.0%)
Missing	19 (24%)	17 (24%)	2 (25%)
TNM Stage			
IA	15 (25.9%)	13 (25.0%)	2 (33.3%)
IB	23 (39.7%)	21 (40.4%)	2 (33.3%)
II	6 (10.3%)	5 (9.6%)	1 (16.7%)
IIIA	1 (1.7%)	1 (1.9%)	0 (0.0%)
IIIB	8 (13.8%)	7 (13.5%)	1 (16.7%)
IV	5 (8.6%)	5 (9.6%)	0 (0.0%)
Missing	20 (26%)	18 (26%)	2 (25%)

ECOG PS, Eastern Cooperative Oncology Group performance status.

a n (%); Mean (SD); y, year; Eastern Cooperative Oncology Group Performance Status (ECOG PS)

### Treatment Patterns

Among the 612 patients treated with first-line PP, 237 patients (39%) did not receive any further systemic treatment. The remaining 375 patients (61%) received second-line systemic therapies. In total, PP rechallenge was administered in 78 patients (21%). The most common regimen was single-agent chemotherapy, administered in 143 patients (38%), with gemcitabine administered in 89, vinorelbine in 53, and cisplatin monotherapy in one patient. Single-agent pemetrexed was administered in 32 patients (8.5%), and platinum combined with another chemotherapy agent in 30 patients (8%), including gemcitabine in 16, vinorelbine in 13, and docetaxel in one. Nivolumab/ipilimumab was administered in 29 patients (7%). Single-agent programmed cell death protein 1 inhibition was administered to 57 patients (15%; nivolumab in 30 and pembrolizumab in 27). Tremelimumab, a cytotoxic T-lymphocyte-associated protein 4 inhibitor, was administered in six patients (2%). The median number of second-line cycles administered was four (IQR: 3–4) in the PP cohort, including 16 patients who received pemetrexed maintenance for a median of three cycles (IQR: 2–4). The median number of second-line cycles administered was four (IQR: 2–5) across all regimens.

### Efficacy of Second-Line Treatment

The median follow-up time was 67.7 months (95% CI: 37.6–not reached [NR]) for patients re-treated with PP and 54.7 months (95% CI: 37.6–75.9) for the whole second-line cohort. The rw-mOS and the rw-mPFS for patients treated only with first-line PP without subsequent second-line treatment were 10.2 months (95% CI: 8.6–11.3) and 6.7 months (95% CI: 6–8), respectively, calculated from the initiation of first-line therapy.

Among all patients treated with any second-line therapy, rw-mOS and rw-mPFS were 11.6 months (95% CI: 9.9–13) and 4.7 months (95% CI: 3.9–5.2), respectively, from second-line initiation, indicating that patients who are fit enough to receive further treatment derive an additional survival benefit. The 1-year, 2-year, and 5-year OS rates were 48%, 22%, and 6%, respectively. The outcomes for the second-line cohort, PP rechallenge, and nivolumab/ipilimumab are summarized in Table 2. The Kaplan-Meier curves for rw-OS and rw-PFS probability across second-line regimens are depicted in Figure 1*A* and *B*. Among these patients, the platinum-sensitive group reported improved rw-mOS (14.8 mo versus 9 mo; HR = 2, 95% CI: 1.6–2.5, *p <* 0.0001) and improved rw-mPFS (6 mo versus 3 mo; HR = 1.6, 95% CI: 1.3–1.9, *p <* 0.0001) compared with the platinum-resistant group.

**Table 2:** Second-Line Treatment Outcomes by Regimen and PFI

Regimen	N	Median OS (mo)	Median PFS (mo)	OS: Sensitive vs. Resistant (mo)	*p* Value	PFI/TFI: Sensitive vs. Resistant (mo)	*p* Value
All 2L regimens	375	11.6	4.7	14.8 vs. 9	*p* < 0.0001	6 vs. 3	*p* < 0.0001
Platinum plus pemetrexed	78	20.5	6.8	21.4 vs. 11.9^a^	*p =* 0.007	7.2 vs. 5.1^a^	*p =* 0.003
Nivolumab plus ipilimumab	29	22.8	8.0	32.3 vs. 18.1	*p =* 0.03	9 vs. 3.8	*p =* 0.02

OS and PFS from 2L initiation: PFI/TFI, sensitive (≥ 6 mo) versus resistant (<6 mo).

2L, second-line; CHT, chemotherapy; OS, overall survival; PFI, platinum-free interval; PFS, progression-free survival; TFI, treatment-free interval.

a Platinum-resistant subgroup, n = 11; interpret with caution given the limited sample size.

**Figure 1:**
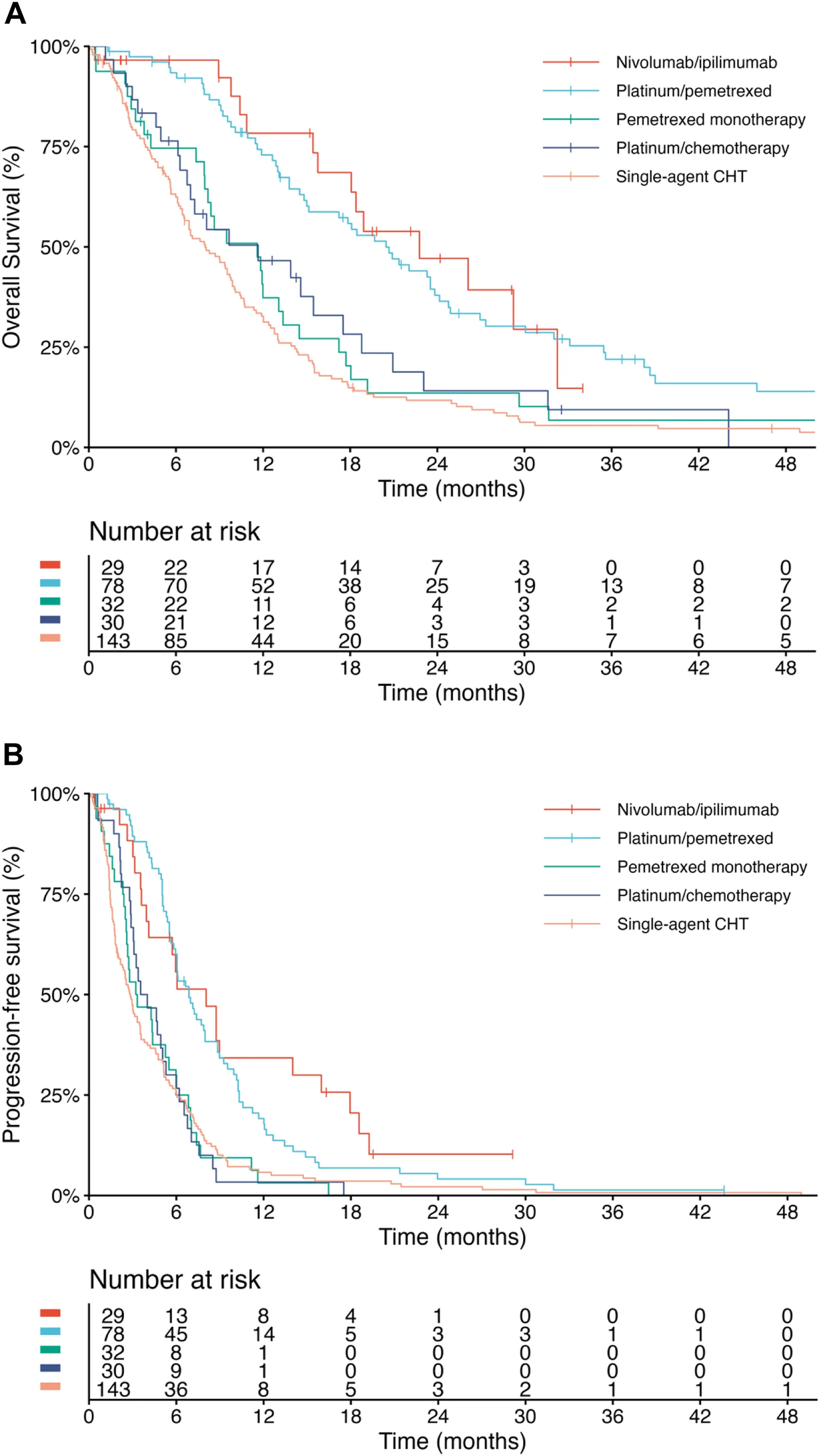
Overall survival *(A)* and real-world progression-free survival *(B)* stratified by second-line regimen. CHT, chemotherapy.

#### Efficacy of PP Rechallenge According to PFI

Among the 78 patients who received second-line PP rechallenge, the rw-mOS and rw-mPFS were 20.5 months (95% CI: 15.0–24.1) and 6.8 months (95% CI: 5.9–8.8), respectively (Fig. 2*A* and *B*). By histologic subtype, the rw-mOS was 20.5 months (95% CI: 15.0–24.1) in patients with the epithelioid subtype and 21.4 months (95% CI: 11.6–NR) in those with the nonepithelioid subtype (Fig. 2*C*). No difference was noted between the groups (HR = 0.8, 95% CI: 0.3–1.8, *p =* 0.5), likely reflecting the low number of patients with the nonepithelioid subtype. Similarly, the rw-mPFS was 6.9 months (95% CI: 5.9–9.0) in those with epithelioid and 6.9 months (95% CI: 5.3–NR) in those with the nonepithelioid subtype (HR = 1, 95% CI: 0.5–2.3, *p =* 0.9) (Fig. 2*D*).

**Figure 2:**
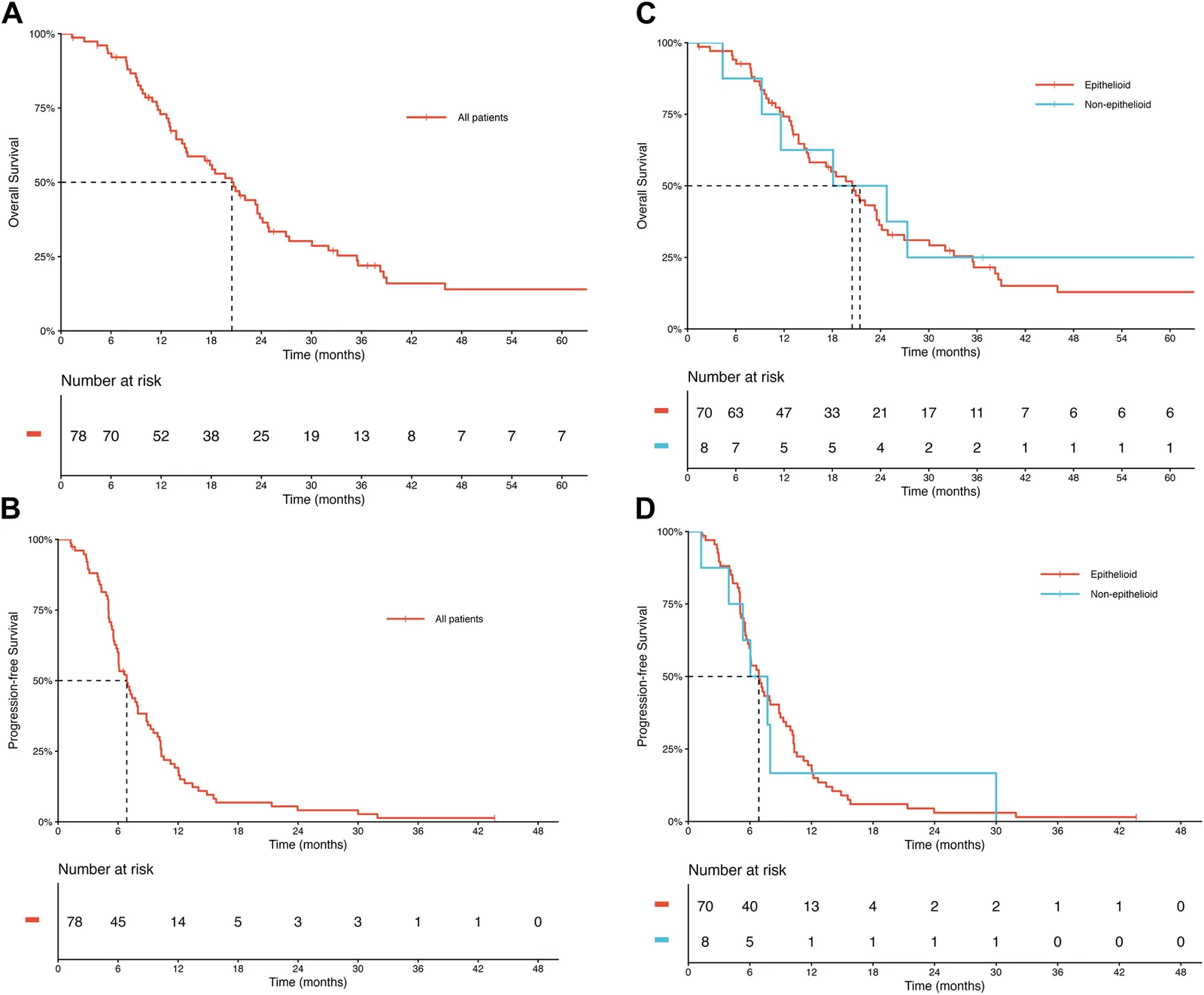

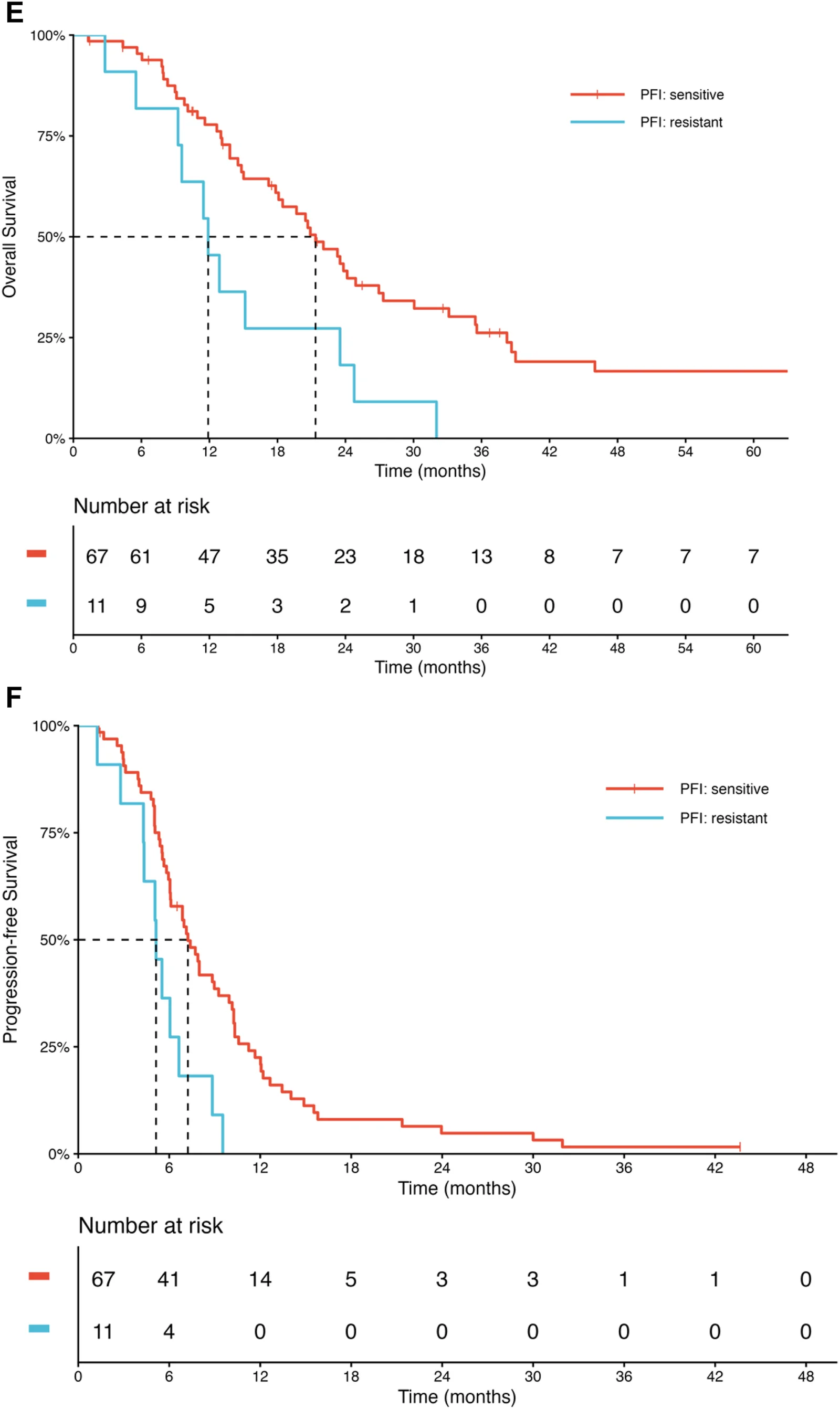
OS and PFS in all patients receiving second-line platinum/pemetrexed rechallenge *(A)* OS and *(B)* PFS; *(C)* OS and (*D*) PFS by histological subtype; and *(E)* OS and *(F)* PFS by PFI category. OS, overall survival; PFI, platinum-free interval; PFS, progression-free survival.

The 1-year, 2-year, and 5-year OS rates were 72%, 37%, and 14%, respectively, from initiation of second-line treatment and 98%, 80%, and 24%, respectively, from the start of first-line treatment, reflecting the OS trajectory from diagnosis. Detailed survival estimates by year and for various clinical covariates are presented in Table 3 and Supplementary Table 2, respectively. Exploratory analysis of OS by TNM stage (n = 58; 20 patients [26%] were excluded because of missing stage data) found no significant difference in rw-mOS across stage groups (IA: 18.1 mo, IB: 21.4 mo, II: 17.2 mo, IIIB: 19.2 mo, IV: 21.6 mo). The single patient with stage IIIA disease had a shorter rw-mOS (6.1 mo), which should be interpreted with caution given its singularity. In a multivariable analysis including subtype, age, ECOG PS, TNM stage, and PFI, the platinum-resistant group remained the only independent prognostic factor for OS (HR = 3.31, 95% CI: 1.3–8.0, *p =* 0.009). Subtype, age, ECOG PS, and TNM stage were not independently associated with OS (Supplementary Table 3). This model was based on a reduced sample because of missing covariate data (n = 52, 43 events; 26 patients were excluded because of missing data), yielding approximately 8.6 events per variable; results should therefore be interpreted with caution.

**Table 3:** Survival Rates Among Patients Treated in the Second-Line Setting by Regimen

Treatment	From Start of:	1-y OS (95% CI)	2-y OS (95% CI)	3-y OS (95% CI)	4-y OS (95% CI)	5-y OS (95% CI)
Any regimen	Second-line	48% (43%–53%)	22% (18%–27%)	12% (8.5%–16%)	8.4% (5.6%–12%)	6.1% (3.7%–10%)
PP rechallenge	First-line	98% (94%–100%)	80% (71%–89%)	55% (45%–67%)	35% (25%–48%)	24% (15%–37%)
	Second-line	72% (63%–83%)	37% (27%–50%)	21% (13%–34%)	14% (7.2%–26%)	14% (7.2%–26%)
	Epithelioid	73% (63%–85%)	36% (26%–50%)	21% (13%–35%)	13% (6.2%–26%)	13% (6.2%–26%)
	Nonepithelioid	67% (42%–100%)	44% (21%–92%)	22% (6.5%–75%)	22% (6.5%–75%)	22% (6.5%–75%)
Nivolumab plus ipilimumab	Second-line	78% (63%–97%)	47% (29%–76%)	15% (2.9%–74%)	15% (2.9%–74%)	15% (2.9%–74%)

CI, confidence interval; OS, overall survival; PP, Platinum/pemetrexed.

Most patients treated with PP rechallenge were platinum-sensitive, with a median PFI of 13.8 months (IQR: 7.9–23.0). Only 11 patients were platinum-resistant. Patients with platinum sensitivity achieved a longer rw-mOS of 21.4 months compared with patients with platinum resistance (11.9 mo; HR = 2.5, 95% CI: 1.3–4.8, *p =* 0.007; Fig. 2*E*). Similarly, rw-mPFS was improved in patients with platinum sensitivity compared with patients with platinum resistance (7.2 mo versus 5.1 mo; HR = 2.6, 95% CI: 1.3–5.2, *p =* 0.003; Fig. 2*F*). In an additional multivariable analysis (Supplementary Table 4), the platinum-resistant group remained the only independent prognostic factor for OS (HR = 2.92, 95% CI: 1.4–5.9, *p =* 0.003). Although the outcome differences across both PFI categories were statistically significant, the CI was wide, reflecting the limited number of patients in the platinum-resistant subgroup, warranting interpretation with caution.

Response data were available for 59 patients (75%) in the second-line and 60 patients (77%) in the first-line. The best objective response in the second-line was a partial response (PR) in 19 patients (32%), and disease control (PR plus stable disease) was reported in 52 patients (88%). For comparison, in the first-line setting, four patients (6%) achieved a complete response, 27 patients (45%) achieved a PR, and 56 patients (93%) achieved disease control.

In univariable analysis, an elevated NLR (≥5; HR = 2.21, 95% CI: 1.3–3.8, *p =* 0.004), an elevated platelet-to-lymphocyte ratio (≥236; HR = 1.79, 95% CI: 1.1–3.0, *p =* 0.027), an elevated systemic immune-inflammation index (≥1270; HR = 2.24, 95% CI: 1.3–3.9, *p =* 0.004), and an intermediate lung immune prognostic index score (HR = 2.32, 95% CI: 1.4–3.9, *p =* 0.002) at the time of diagnosis were each associated with shorter OS among patients receiving PP rechallenge. None retained independent prognostic significance in multivariable analysis. In contrast, none of these biomarkers had an association with OS in univariable or multivariable analysis among patients treated with second-line nivolumab/ipilimumab (Supplementary Table 4).

#### Efficacy of Second-Line Nivolumab/Ipilimumab According to PFI

Among the 29 patients who received second-line nivolumab/ipilimumab, the rw-mOS and rw-mPFS were 22.8 months (95% CI: 18.1–NR) and 8.0 months (95% CI: 4.1–17.9), respectively (Fig. 1*A* and *B*). Both OS and rw-mPFS were comparable to the outcomes observed in the PP rechallenge subcohort (OS: HR = 1.12, 95% CI: 0.6–2.0, *p =* 0.7; rwPFS: HR = 1.3, 95% CI: 0.8–2.1, *p =* 0.3). The 1-year, 2-year, and 5-year OS rates were similar to those with PP rechallenge (78%, 47%, and 15%, respectively, from initiation of second-line treatment). By histology, the rw-mOS was 18.9 months in epithelioid and 22.8 months in nonepithelioid subtype (HR = 0.9, 95% CI: 0.3–3.0, *p =* 0.84), and the rw-mPFS was 6 months in epithelioid and 8.9 months in nonepithelioid subtype (HR = 0.6, 95% CI: 0.2–1.6, *p =* 0.33), without statistically detectable differences between the groups.

Ten patients (34%) were platinum-resistant, and the median TFI was 6.9 months (IQR: 4.8–11.0); TFI was associated with improved rw-mOS and rw-mPFS. Patients with platinum sensitivity had a median OS of 32.3 months, compared with 18.1 months in patients with platinum resistance (HR = 3, 95% CI: 1–9.2, *p =* 0.03). Similarly, median rw-mPFS was 9 months in patients with platinum sensitivity and 3.8 months in patients with platinum resistance (HR = 3, 95% CI: 1.2–8, *p =* 0.02).

#### Efficacy of Other Second-Line Regimens

Among 32 patients who received single-agent pemetrexed in the second-line setting, rw-mOS and rw-mPFS were 11.6 months (95% CI: 8.0–14.5) and 3.3 months (95% CI: 2.6–6.0), respectively (Fig. 1*A* and *B*). By histology, the epithelioid subtype was associated with longer rw-mOS and rw-mPFS compared with the nonepithelioid subtype (rw-mOS: 11.6 mo versus 7.6 mo, HR = 1.1, 95% CI: 0.5–2.7, *p =* 0.76; and rw-mPFS: 4.3 months versus 2.7 months, HR = 1.5, 95% CI: 0.6–3.3, *p =* 0.37), without a significant difference between the groups. Single-agent pemetrexed had significantly worse rw-mOS and rw-mPFS compared with PP rechallenge (rw-mOS: HR = 0.43, 95% CI: 0.3–0.7, *p =* 0.0001; rw-mPFS: HR = 0.43, 95% CI: 0.3–0.6, *p =* 0.0001). We also sought to evaluate the outcome differences between PP rechallenge and single-agent pemetrexed in patients with TFI of 6 months or longer. Among 67 PP rechallenge patients with platinum-sensitivity and 24 single-agent pemetrexed patients with platinum-sensitivity, each with TFI of 6 months or longer, PP rechallenge seemed to improve both rw-mOS (21.4 mo versus 11.6 mo, HR = 0.42, 95% CI: 0.2–0.7, *p <* 0.0001) and rw-mPFS (7.2 mo versus 3.5 mo, HR = 0.42, 95% CI: 0.3–0.7, *p =* 0.0003) compared with single-agent pemetrexed.

Among patients receiving platinum-based chemotherapy (comprising nonpemetrexed chemotherapy), the rw-mOS was 11.7 months (95% CI: 7.0–18.8); single-agent immune checkpoint-inhibitor (ICI) yielded 10.7 months (95% CI: 8.2–18.8), and single-agent chemotherapy achieved 8.0 months (95% CI: 6.5–9.9). With regard to rw-mPFS, single-agent ICI yielded a rw-mPFS of 6.6 months (95% CI: 3.5–8.2), which was longer than that with other chemotherapy regimens (*p =* 0.001): platinum/chemotherapy achieved a median rw-mPFS of 3.8 months, single-agent pemetrexed achieved 3.3 months, and single-agent chemotherapy achieved 2.8 months.

Six patients who received tremelimumab monotherapy had a rw-mOS of 3.4 months (95% CI: 1.7–NR) and a rw-mPFS of 1.7 months, which is consistent with the limited activity of single-agent CTLA-4 inhibitors in PM.

#### Treatment Attrition

Among 612 patients treated with first-line PP, 237 patients (39%) did not receive any further systemic treatment. Of these, 57 died within 3 months of treatment initiation, and an additional 58 patients died within the subsequent three months, accounting for 48% of deaths within 6 months of starting first-line therapy. In 108 patients, first-line treatment was discontinued early; reported reasons were adverse events in 41 patients, progressive disease in 41, patient preference in four, and unknown reasons in 22. The remaining 129 patients completed the planned first-line treatment cycles.

#### Third-Line Treatment

Among the 375 patients who received second-line therapy, 194 patients (52%) went on to receive third-line treatment (Fig. 3). Receipt of third-line therapy was strongly associated with improved rw-mOS from second-line initiation compared with nonreceipt of further treatment (17.2 mo versus 6.3 mo; HR = 0.4, 95% CI: 0.3–0.5, *p <* 0.0001). The most typically administered regimens were single-agent chemotherapy in 89 patients (46%; vinorelbine in 53 and gemcitabine in 36), single-agent ICI in 40 patients (21%; nivolumab in 24 and pembrolizumab in 16), platinum-based chemotherapy (PP) in 20 patients (10%), single-agent pemetrexed in 18 patients (9%), nivolumab plus ipilimumab in 17 patients (9%), platinum combined with other chemotherapy agents in seven patients (4%), and gemcitabine plus ramucirumab in three patients (2%).

**Figure 3:**
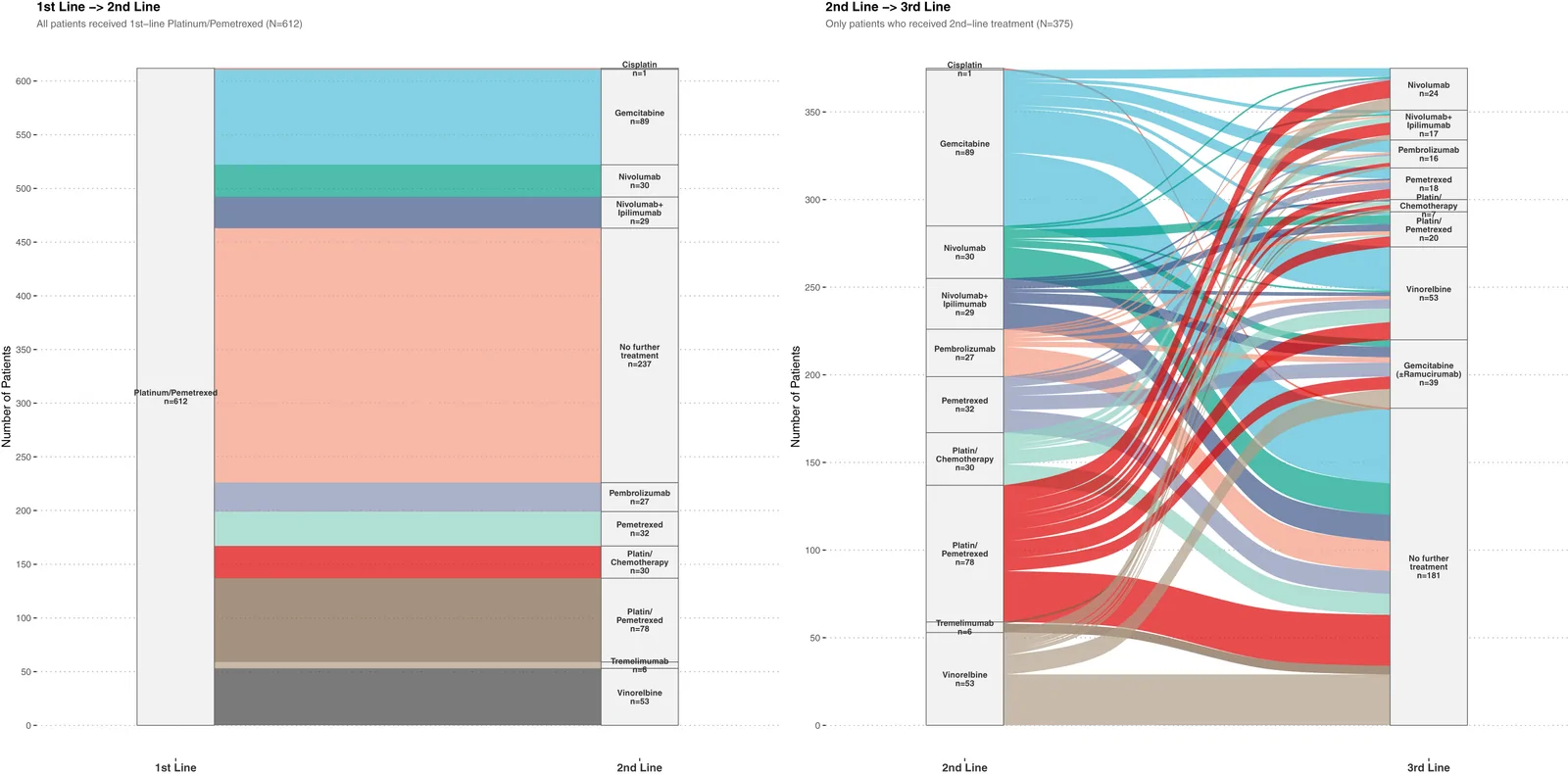
Treatment patterns and attrition in first-line, second-line, and third-line settings.

Among the 78 patients who received second-line PP rechallenge, 49 patients (63%) went on to receive third-line systemic therapy (Fig. 3). Receipt of third-line therapy was strongly associated with improved rw-mOS from second-line initiation compared with patients who did not receive further treatment (23.8 mo versus 11.6 mo; HR = 0.4, 95% CI: 0.2–0.7, *p <* 0.001). The most common regimens were single-agent chemotherapy in 17 patients (35%; vinorelbine in 10 and gemcitabine in seven), single-agent ICI in 12 patients (24%; nivolumab in 10 and pembrolizumab in two), and nivolumab plus ipilimumab in seven patients (14%). Further rechallenge with PP was administered in six patients (12%), single-agent pemetrexed in five (10%), and platinum combined with other chemotherapy agents in two (4%).

Among a very small subset of four patients who received third-line PP after second-line nivolumab plus ipilimumab, the rw-mOS was 29.2 months (95% CI: 10.9–NR) from second-line initiation and 12.4 months (95% CI: 4.3–NR) from third-line initiation, with a rw-mPFS of 11.8 months (95% CI: 3.1–NR) and 5.3 months (95% CI: 4.2–NR), respectively.

## Discussion

This real-world analysis of patients with PM treated with first-line PP represents one of the largest cohorts to date examining second-line treatment patterns and outcomes in this disease. Approximately 61% of patients were treated with second-line therapy, of whom around one-third received pemetrexed-based rechallenge—either in combination with platinum (PP rechallenge) (21%) or single-agent pemetrexed (8.5%). Despite the approval of nivolumab plus ipilimumab, the paucity of effective treatment options underscores the importance of optimal sequencing of available regimens, specifically of pemetrexed-based regimens. Although rechallenge with nivolumab plus ipilimumab may carry an increased risk after immune-related toxicity, as previously reported in PM, pemetrexed rechallenge reflects routine real-world practice.^28^ The guideline recommendation supporting pemetrexed-based rechallenge has been based on small retrospective studies of not more than 31 patients treated with each specific regimen and heterogeneous populations.^21^^,^^22^^,^^25^^,^^29^ In addition, a meta-analysis of 49 studies on second-line treatment concluded an objective response rate of 30% with PP rechallenge and 18% with pemetrexed, and a median OS of 8 months for both regimens.^30^ Our cohort substantially extends this evidence, and when contemporary therapies are taken into account, broadly confirms that PP rechallenge is an active regimen in selected patients and supports the use of PP over single-agent pemetrexed for improved outcomes. With a rw-mOS of 20.5 months and a disease control rate of 88%, which are comparable to those achieved in the recent first-line trial,^3^ our study suggests retained PP sensitivity in carefully selected patients.

The median PFI of 13.8 months in our PP rechallenge subcohort indicates that most re-treated patients were platinum-sensitive and most of them had the epithelioid subtype, likely contributing to the favorable outcomes observed. Nonetheless, the PFI analysis found a longer rw-mOS in patients with platinum sensitivity (21.4 mo) than in those with platinum resistance (11.9 mo), suggesting PFI’s potential role as a meaningful selection criterion. This was further corroborated in multivariable analysis, in which platinum resistance was found to be the only independent prognostic factor for OS (HR = 2.9, 95% CI: 1.4–5.9, *p =* 0.003). Although small numbers preclude definitive conclusions, even patients with platinum-resistant disease achieved a rw-mOS of 11.9 months, suggesting that PP rechallenge may offer potential benefit even beyond the platinum-sensitive population, though this warrants further validation.

Whether the addition of a platinum compound to pemetrexed provides benefit over monotherapy has been unclear. In previous findings, Zucali et al.^25^ reported a substantial OS benefit with platinum addition (13.4 mo versus 4.2 mo among 31 [PP] and 11 patients [pemetrexed only], respectively), whereas Bearz et al.^21^ found no significant difference when including only patients with a time to progression of more than 6 months. To further address this question, we compared outcomes between PP rechallenge and single-agent pemetrexed, which confirmed a significant survival benefit of PP rechallenge over single-agent pemetrexed, including among patients with platinum sensitivity. Although this survival benefit is compelling, it should be interpreted with caution as it may be confounded by patient selection, given that fitter and less comorbid patients are more likely to be offered platinum-pemetrexed. Notably, the PP rechallenge subcohort had a high proportion of early-stage disease, likely reflecting the selection of patients fit for rechallenge. Nevertheless, we found no evidence of improved survival among patients with early-stage disease. Furthermore, the platinum-resistant group remained the only independent predictor of OS in a multivariable analysis adjusting for subtype, age, ECOG PS, and TNM stage, suggesting that the favorable outcomes observed are not simply explained by these baseline clinical characteristics, though a high proportion of missing data and small subgroup sizes limit definitive conclusions. Furthermore, receipt of third-line therapy was strongly associated with improved rw-mOS in the PP rechallenge subcohort (23.8 mo versus 11.6 mo; HR = 0.39, 95% CI: 0.23–0.68, *p <* 0.001), likely reflecting patient selection bias.

First-line PP remains a viable alternative to nivolumab plus ipilimumab, particularly in the epithelioid subtype, in a resource-restricted setting, and when ICI is contraindicated. Our unpublished data report that around 18% of patients in Germany received first-line PP since the European Medicines Agency’s approval of nivolumab plus ipilimumab, and 37% of them had the non-epithelioid subtype, suggesting that a substantial proportion of patients continues to receive first-line PP and will potentially be candidates for PP rechallenge. Among other second-line regimens, outcomes achieved with nivolumab plus ipilimumab were comparable to those with PP rechallenge (rw-mOS 22.8 mo, rw-mPFS 8.0 mo) and were consistent with published phase II data when accounting for the inclusion of patients treated with multiple prior lines of treatment. Similarly, the one-year OS in our cohort (78%) exceeded that reported in the INITIATE trial (64%) and the MAPS2 trial (58%), both of which evaluated nivolumab/ipilimumab in the later-line setting, likely reflecting the small number of patients in our cohort.^31^^,^^32^ Notably, nivolumab plus ipilimumab yielded a numerically higher median OS in both patients with platinum sensitivity (32.3 months) and patients with platinum resistance (18.1 mo) compared with PP rechallenge in the respective settings, suggesting it may offer a benefit regardless of TFI status. The observation that PFI and TFI were similarly prognostic for outcomes with both PP rechallenge and nivolumab/ipilimumab suggests that PFI or TFI may reflect broader tumor biology and disease aggressiveness rather than platinum-specific chemosensitivity. Nevertheless, given the small subgroup of patients treated with nivolumab/ipilimumab (platinum-sensitive, n = 19; platinum-resistant, n = 10), these findings should be interpreted cautiously.

Currently, nivolumab/ipilimumab is preferred as a first-line treatment, particularly in nonepithelioid histology. Therefore, in the current scenario, PP rechallenge may become relevant in the third-line setting among patients who are treated with first-line nivolumab plus ipilimumab followed by second-line PP, a sequence that, to our knowledge, has not been evaluated yet. Our preliminary data from four patients suggest that PP rechallenge may retain activity even after second-line nivolumab plus ipilimumab. Nevertheless, in patients who receive PP as first-line treatment, second-line nivolumab plus ipilimumab may represent a reasonable option, given the meaningful efficacy irrespective of TFI in this small subgroup.

In our cohort, single-agent ICI, single-agent pemetrexed, and platinum-based chemotherapy achieved rw-mOS between 10 and 11 months, consistent with the phase III PROMISE-Meso and CONFIRM trials and prior real-world reports.^33^, ^34^, ^35^, ^36^ Nevertheless, because our patients were ICI-naïve, the applicability of these findings in the current era, when nivolumab/ipilimumab represents the preferred first-line regimen, remains uncertain.

The prognostic role of inflammatory biomarkers has been previously reported in PM.^26^^,^^37^ In our analysis, peripheral blood inflammatory biomarkers were associated with OS in univariable but not multivariable analyses among patients receiving PP rechallenge. No association was observed in the nivolumab/ipilimumab group, although the small sample size limits interpretation.

In our cohort, 61% of patients received second-line treatment after first-line PP, which compares favorably to the historic EAP program (42%) conducted after the EMPHACIS trial, and probably reflects the incorporation of ICI into the treatment landscape (14% in our cohort).^38^ Nevertheless, eligible patients, when carefully guided to further treatment options, may achieve improved survival.

This study has several limitations. Its retrospective design introduces selection bias. Treatment decisions were not randomized and reflected the physician’s judgment regarding patient fitness and disease characteristics. The timing and evaluation of tumor response were conducted according to local standards, which may have influenced our response analyses. Small subgroup sizes, particularly in patients with platinum resistance, limit appropriate and meaningful subgroup analyses. In addition, patients with sarcomatoid histology were not represented in the PP rechallenge subcohort. Patients with sarcomatoid histology in our cohort generally experienced a short time to second-line treatment (median, 2.7 mo, IQR: 1.8–4.6), likely reflecting poorer chemotherapy responsiveness and limiting eligibility for rechallenge strategies. Consequently, our findings on PFI-guided PP rechallenge are not generalizable to sarcomatoid histology. Nonetheless, given the exploratory nature of this study, prospective evaluation in larger cohorts is warranted.

## Conclusion

Platinum plus pemetrexed rechallenge was the most typically used pemetrexed-based second-line regimen and had favorable outcomes in selected patients, with a rw-mOS of 20.5 months and a disease control rate of 88%. PFI was associated with clinically meaningful differences in outcomes, with patients with platinum sensitivity achieving a rw-mOS of 21.4 months with PP rechallenge. Nevertheless, nivolumab plus ipilimumab had numerically superior outcomes in both TFI categories, suggesting that it may offer benefit regardless of TFI status. In patients with platinum sensitivity, PP rechallenge remains a viable alternative, particularly where immunotherapy is contraindicated or unavailable. In patients with platinum resistance, nivolumab/ipilimumab may be considered, given the limited benefit observed with PP rechallenge in this group. Prospective evaluation of PFI or TFI-guided second-line treatment selection is warranted to formally establish optimal treatment sequencing in PM.

## CRediT Authorship Contribution Statement

**Rajiv Shah:** Conceptualization, Data curation, Formal analysis, Methodology, Visualization, Writing - original draft, Writing - review & editing.

**Martin Reck:** Conceptualization, Methodology, Writing - original draft, Writing - review & editing.

**Christian Grohé:** Conceptualization, Methodology, Writing - original draft, Writing - review & editing.

**Martin Faehling:** Conceptualization, Methodology, Writing - original draft, Writing - review & editing.

**Daniel C. Christoph:** Conceptualization, Methodology, Writing - original draft, Writing - review & editing.

**Felix C. Saalfeld:** Conceptualization, Methodology, Writing - original draft, Writing - review & editing.

**Eva L. Buchmeier:** Conceptualization, Methodology, Writing - original draft, Writing - review & editing.

**Hans-Georg Kopp:** Conceptualization, Methodology, Writing - original draft, Writing - review & editing.

**Petra Hoffknecht:** Conceptualization, Methodology, Writing - original draft, Writing - review & editing.

**Nikolaj Frost:** Conceptualization, Methodology, Writing - original draft, Writing - review & editing.

**Frank Griesinger:** Conceptualization, Methodology, Writing - original draft, Writing - review & editing.

**Jonas Kuon:** Conceptualization, Methodology, Writing - original draft, Writing - review & editing.

**Jingting Luan:** Data curation.

**Julia Roeper:** Data curation.

**Martin Kimmich:** Data curation, Writing - original draft.

**Till Olchers:** Data curation.

**Paolo Chesi:** Data curation.

**Laura V. Klotz:** Conceptualization, Methodology, Writing - original draft.

**Inn Chung:** Data curation, Project administration.

**Helge Bischoff:** Writing - original draft, Writing - review & editing.

**Michael Thomas:** Conceptualization, Methodology, Writing - original draft, Writing - review & editing.

**Petros Christopoulos:** Conceptualization, Data curation, Formal analysis, Methodology, Writing - original draft, Writing - review & editing.

## Disclosure

Dr. Shah has received a research grant for an investigator-initiated trial from Bristol Myers Squibb (BMS) (institutional), compensation for speakers’ bureau and consulting fees from AstraZeneca (AZ), Novartis, SanofiAventis, Johnson&Johnson, MSD, neoConnect and Roche; has received support for attending meetings and travel from AZ, Johnson&Johnson, Lilly and Pfizer; has received honoraria for advisory board meetings from Johnson&Johnson; holds a leadership role at the pulmo-oncology working group of the German cancer society. Prof. Dr. Reck has received consulting fees, speakers’ bureau fees, and meeting and travel support from Amgen, AZ, Beigene, Boehringer-Ingelheim (BI), BMS, Lilly, Merck, MSD, Mirati, Novartis, GSK, Pfizer, Roche, Regeneron, Sanofi, Daiichi-Sankyo (DS), and Janssen; has been compensated for data safety board from Sanofi, DS, and Servier; has received honoraria from Accord. Prof. Dr. Grohé has received speaker’s fees, consulting fees, and travel support from AZ, BMS, BI, and DS. Dr. Faehling has received consulting fees from Roche, BMS, MSD, AZ, and research grants to institution from Roche, BMS, MSD, AZ. Dr. Christoph has received consulting fees, speakers’ bureau fees, expert testimony fees, compensation for data safety board from Amgen, AZ, BMS, DS, Johnson&Johnson, Lilly, Merck, MSD, Novocure, Pfizer, PharmaMar, Pierre Fabre, Regeneron, Roche, and Sanofi; has received support from attending meetings and travel from AZ, Johnson&Johnson, Lilly, Novocure, Merck, MSD, Pfizer, PharmaMar, and Roche. Dr Saalfeld has received grants from BI and Roche; has received fees for speaker’s bureau from Janssen, Takeda, BI, Roche, Novartis, Thieme, GWT-TUD, Lilly, Amgen, AZ, Pfizer, Accord, CME Verlag, support for meetings and travels from AZ, Johnson&Johnson, Lilly, and BI; has been compensated for advisory meetings from Janssen, BI, Pfizer, Roche, BeOne, Amgen, BMS, AZ, MSD, BMS, and DS; holds a leadership role at the Working group Medical Oncology-Committee on Thoracic Oncology of the German Cancer Society. Dr. Buchmeier has received consulting fees from Janssen, AZ, BMS, and Takeda and travel support from Janssen. Prof. Dr. Kopp has received support for manuscript from MSD, BMS, Roche and AZ, consulting fees from MSD, BMS, Roche, and AZ, honoraria from MSD, BI, Roche, Pfizer, Takeda, Novartis, BMS, SanofiAventis, AZ and Lilly, meeting and travel support from Johnson&Johnson, Pfizer, Amgen, and MSD; has received compensations for advisory board meetings from MSD, BMS, Roche, AZ, Johnson&Johnson. Dr. Hoffknecht has been compensated for data safety board from AZ, MSD, Johnson&Johnson, Lilly, BMS, and DS. Dr. Frost has received research personal and institutional grant from Roche, fees for advisory board meetings from Abbvie, Amgen, Astellas, AZ, BeiGene, BMS, BI, Chugai, DS, Eisai, Eli Lilly, Genmab, GSK, Janssen, Merck, MSD, Novartis, Pfizer, Roche, Takeda, fees for speaker’s bureau from Amgen, AZ, BerlinChemie, BI, MSD, Novocure, Pierre-Fabre, Regeneron, Roche, Sanofi, and consulting fee from MSD and Roche; holds a leadership role at the German Society of Pneumology and Working Group of thoracic oncology of German cancer society. Prof. Dr. Griesinger has received fees for speakers’ bureau and advisory board meetings and holds other financial or nonfinancial interests from AZ, BI, BMS, Celgene, Lilly, MSD, Novartis, Pfizer, Roche, Takeda, Ariad, Abbvie, and Siemens. Ms. Luan has received the AIO scholarship 2023, funded by an unrestricted grant by donations from Amgen, AZ, DS, Janssen, Roche and Takeda, and honoraria from DSHO 2023. Dr. Kimmich has received fees for speakers’ bureau and consulting from AZ and Pierre-Fabre, meeting and travel support from Pierre-Fabre and BeOne, and advisory board from Janssen, Johnson&Johnson, Amgen, and Pierre-Fabre. Prof. Dr. Thomas has received research grant to institution from AZ, BMS, Johnson&Johnson, Merck, Pharmamar, Roche, Takeda, fee for consulting and speakers’ bureau from Amgen, AZ, Beigene, BMS, BI, DS, Gilead Sciences, GSK, Johnson&Johnson, Lilly, Merck, MSD, Novartis, Pfizer, Pharmamar, Pierre Fabre, Regeneron, Roche, Sanofi, Takeda and meetings and travels support from AZ, BMS, BI, DS, Johnson&Johnson, Lilly, Merck, MSD, Novartis, Pfizer, Pharmamar, Pierre Fabre, Roche, Sanofi, and Takeda. Prof. Dr. Christopoulos has received research grant to institution from Roche, Amgen, BI, Takeda, Merck, AZ, Novartis, fees for speakers’ bureau from Roche, Takeda, AZ, Gilead, Merck, Thermo-Fisher, Janssen, Pfizer, Novartis, and BeOne, meetings and travels support from AZ, Pfizer, Janssen, Merck, Gilead, DS, Takeda, Novartis, Lilly and fee for advisory board meetings from Pfizer, Chugai, BI, Takeda, Janssen, Novartis, AZ, MSD, Roche. The remaining authors declare no conflict of interest.

## Footnotes

Note: To access the supplementary material accompanying this article, visit the online version of the *JTO Clinical and Research Reports* at www.jtocrr.org and at https://doi.org/10.1016/j.jtocrr.2026.101073.
