# Supplementary material for: Outcomes of Platinum plus /Pemetrexed Rechallenge in Pleural Mesothelioma: The Role of Platinum-Free Interval in a Multicenter Real-World Analysis
Source: JTO Clin Res Rep. 2026 Aug 31;7(11):101073. doi: 10.1016/j.jtocrr.2026.101073 (PMC13742269; doi:10.1016/j.jtocrr.2026.101073)
Supplement: Supplementary file 1 [file mmc1.docx]

Supplementary Table

Table S1. Patient characteristics of overall first-line platinum/pemetrexed cohort

|  | Overall  N = 612^1^ | Epithelioid  N = 463^1^ | Non-epithelioid  N = 149^1^ |
| --- | --- | --- | --- |
| **Sex** |  |  |  |
| *Male* | 505 (82.5%) | 378 (81.6%) | 127 (85.2%) |
| *Female* | 107 (17.5%) | 85 (18.4%) | 22 (14.8%) |
| **Age (Mean)** | 70 (9) | 69 (9) | 70 (9) |
| **Age** |  |  |  |
| *< 65* | 160 (26.1%) | 121 (26.1%) | 39 (26.2%) |
| *≥ 65 to <75* | 273 (44.6%) | 208 (44.9%) | 65 (43.6%) |
| *≥ 75* | 179 (29.2%) | 134 (28.9%) | 45 (30.2%) |
| **Histology** |  |  |  |
| *Epithelioid* | 463 (75.7%) | 463 (100.0%) | 0 (0.0%) |
| *Biphasic* | 92 (15.0%) | 0 (0.0%) | 92 (61.7%) |
| *Sarcomatoid* | 34 (5.6%) | 0 (0.0%) | 34 (22.8%) |
| *Other* | 23 (3.8%) | 0 (0.0%) | 23 (15.4%) |
| **Smoking history** |  |  |  |
| *Never* | 145 (36.0%) | 110 (36.1%) | 35 (35.7%) |
| *Current* | 67 (16.6%) | 53 (17.4%) | 14 (14.3%) |
| *Former* | 191 (47.4%) | 142 (46.6%) | 49 (50.0%) |
| *Missing* | 209 (34%) | 158 (34%) | 51 (34%) |
| **Asbestos exposure** | 340 (77.6%) | 261 (77.2%) | 79 (79.0%) |
| *Missing* | 174 (28%) | 125 (27%) | 49 (33%) |
| **ECOG PS** |  |  |  |
| *0* | 201 (42.2%) | 159 (43.1%) | 42 (39.3%) |
| *1* | 258 (54.2%) | 196 (53.1%) | 62 (57.9%) |
| *≥ 2* | 17 (3.6%) | 14 (3.8%) | 3 (2.8%) |
| *Missing* | 136 (22%) | 94 (20%) | 42 (28%) |
| **TNM Stage** |  |  |  |
| *IA* | 99 (23.3%) | 79 (23.9%) | 20 (21.5%) |
| *IB* | 152 (35.8%) | 118 (35.6%) | 34 (36.6%) |
| *II* | 39 (9.2%) | 31 (9.4%) | 8 (8.6%) |
| *IIIA* | 23 (5.4%) | 19 (5.7%) | 4 (4.3%) |
| *IIIB* | 74 (17.5%) | 53 (16.0%) | 21 (22.6%) |
| *IV* | 37 (8.7%) | 31 (9.4%) | 6 (6.5%) |
| *Missing* | 188 (31%) | 132 (29%) | 56 (38%) |
| **Second-line** |  |  |  |
| *Nivolumab Ipilimumab* | 29 (7.7%) | 20 (6.6%) | 9 (12.5%) |
| *Platin Pemetrexed* | 78 (20.8%) | 70 (23.1%) | 8 (11.1%) |
| *Platin Chemotherapy* | 30 (8.0%) | 23 (7.6%) | 7 (9.7%) |
| *Nivolumab* | 30 (8.0%) | 24 (7.9%) | 6 (8.3%) |
| *Pembrolizumab* | 27 (7.2%) | 18 (5.9%) | 9 (12.5%) |
| *Cisplatin* | 1 (0.3%) | 0 (0.0%) | 1 (1.4%) |
| *Pemetrexed* | 32 (8.5%) | 24 (7.9%) | 8 (11.1%) |
| *Gemcitabine* | 89 (23.7%) | 76 (25.1%) | 13 (18.1%) |
| *Vinorelbine* | 53 (14.1%) | 45 (14.9%) | 8 (11.1%) |
| *Tremelimumab* | 6 (1.6%) | 3 (1.0%) | 3 (4.2%) |
| *none* | 237 (39%) | 160 (35%) | 77 (52%) |
| ^1^n (%); Mean (SD); ECOG PS, eastern cooperative oncology group performance status | | | |

**Table S2. Overall survival and progression-free survival estimates for patients treated with second-line platinum/pemetrexed**

|  | | Overall Survival (OS) | | | Progression-Free Survival (PFS) | | |
| --- | --- | --- | --- | --- | --- | --- | --- |
| **Characteristic** | **N**^1^ | **1-y OS** | **Median OS (mo)** | **p-value**^2^ | **1-y PFS** | **Median PFS (mo)** | **p-value**^2^ |
| Sex |  |  |  | 0.5 |  |  | 0.3 |
| Male | 60 | 71% | 18.5 (15–24) |  | 20% | 7.0 (5.8–9.3) |  |
| Female | 18 | 78% | 23.8 (18–NR) |  | 17% | 6.4 (4.3–10) |  |
| Subtype |  |  |  | 0.6 |  |  | >0.9 |
| Epithelioid | 70 | 74% | 20 .5(15–24) |  | 19% | 6.9 (6.0–9.0) |  |
| Non-epithelioid | 8 | 63% | 21.4 (12–NR) |  | 17% | 6.9 (5.3–NR) |  |
| ECOG PS |  |  |  | 0.15 |  |  | 0.9 |
| 0 | 25 | 70% | 17.2 (14–25) |  | 14% | 7.4 (5.0–11) |  |
| 1 | 34 | 67% | 19.7 (13–33) |  | 15% | 6.1 (5.5–9.5) |  |
| PFI |  |  |  | 0.5 |  |  | 0.14 |
| sensitive (≥ 6m) | 67 | 78% | 21.4 (18–27) |  | 22% | 7.2 (6.1–9.3) |  |
| resistant (< 6m) | 11 | 45% | 11.9 (9.6–NR) |  | 0% | 5.1 (4.3–NR) |  |
| First-line PFS |  |  |  | 0.007 |  |  | 0.004 |
| Good (≥ 6m) | 72 | 75% | 20.9 (18–27) |  | 21% | 7.2 (6.1–10) |  |
| Poor (< 6m) | 6 | 50% | 11.1 (2.8–NR) |  | 0% | 5.1 (4.3–NR) |  |
| Third-line |  |  |  | <0.001 |  |  | 0.7 |
| None | 29 | 43% | 11.6 (9.8–20) |  | 21% | 6.1 (5.3–10) |  |
| Yes | 49 | 88% | 23.8 (21–33) |  | 18% | 7.4 (6.1–10) |  |
| TNM stage |  |  |  | 0.2 |  |  | >0.9 |
| IA | 15 | 70% | 18.1 (12–NR) |  | 21% | 6.7 (5.1–13) |  |
| IB | 23 | 68% | 21.4 (13–32) |  | 23% | 6.1 (5.3–11) |  |
| II | 6 | 67% | 17.2 (10–NR) |  | 0% | 6.9 (5.0–NR) |  |
| IIIA | 1 | 0% | 6.1 (NR–NR) |  | 0% | 6.1 (NR–NR) |  |
| IIIB | 8 | 63% | 19.2 (9.2–NR) |  | 25% | 8.0 (3.0–NR) |  |
| IV | 5 | 100% | 21.6 (14–NR) |  | 0% | 8.9 (5.7–NR) |  |
| ^1^n, ^2^Log-rank test, | | | | | | | |

NR, Not reached; ECOG PS, eastern cooperative oncology group performance status; PFI, platinum-free interval

**Table S3. Univariable and multivariable analyses in the Platinum/Pemetrexed subcohort**

| OS |  | N=78 | HR (univariable) | HR (multivariable) |
| --- | --- | --- | --- | --- |
| **Subtype** | Epithelioid | 70 (89.7) | - | - |
|  | Non-epithelioid | 8 (10.3) | 0.79 (0.34-1.84, p=0.583) | 1.07 (0.38-3.02, p=0.905) |
| **Age (years)** | < 65 | 21 (26.9) | - | - |
|  | ≥ 65 | 57 (73.1) | 1.05 (0.61-1.82, p=0.850) | 1.66 (0.80-3.42, p=0.170) |
| **ECOG PS** | 0 | 25 (42.4) | - | - |
|  | 1 | 34 (57.6) | 0.65 (0.36-1.17, p=0.150) | 0.54 (0.27-1.10, p=0.088) |
| **TNM Stage*** | early | 44 (75.9) | - | - |
|  | late | 14 (24.1) | 1.14 (0.59-2.21, p=0.700) | 1.01 (0.48-2.11, p=0.987) |
| **PFI** | sensitive | 67 (85.9) | - | - |
|  | resistant | 11 (14.1) | 2.46 (1.26-4.83, **p=0.009**) | 3.31 (1.35-8.09, **p=0.009**) |

OS, overall survival; HR, hazard ratio; ECOG PS, eastern cooperative oncology group performance status; PFI, platinum-free interval. *Due to a single value, TNM stage IIIA was excluded.

**Table S4. Univariable and multivariable analyses in the second-line setting**

|  |  | platinum/pemetrexed rechallenge | | | nivolumab/ipilimumab | | |
| --- | --- | --- | --- | --- | --- | --- | --- |
| **OS** |  | **N=78** | HR (univariable) | HR (multivariable) | **N=29** | HR (univariable) | HR (multivariable) |
| **NLR** | < 5 | 49 (62.8) | - | - | 20 (69.0) | - | - |
|  | ≥ 5 | 29 (37.2) | 2.21 (1.28–3.80, **p=0.004**) | 1.26 (0.51-3.16, p=0.616) | 9 (31.0) | 1.72 (0.48–6.16, p=0.408) | 2.03 (0.30-13.62, p=0.466) |
| **PLR** | < 236 | 40 (51.3) | - | - | 16 (55.2) | - | - |
|  | ≥ 236 | 38 (48.7) | 1.79 (1.07–3.01, **p=0.027**) | 0.94 (0.40-2.17, p=0.877) | 13 (44.8) | 0.39 (0.09–1.78, p=0.224) | NE |
| **SII** | < 1,270 | 44 (56.4) | - | - | 21 (72.4) | - | - |
|  | ≥ 1,270 | 34 (43.6) | 2.24 (1.29–3.88, **p=0.004**) | 1.63 (0.53-5.07, p=0.396) | 8 (27.6) | 0.89 (0.19–4.22, p=0.887) | NE |
| **LIPI** | good | 42 (53.8) | - | - | 23 (79.3) | - | - |
|  | intermediate | 33 (42.3) | 2.32 (1.37–3.93, **p=0.002**) | 1.79 (0.91-3.53, p=0.091) | 5 (17.2) | 1.07 (0.29–3.95, p=0.923) | 1.21 (0.29-5.08, p=0.799) |
|  | poor | 3 (3.8)* | 1.60 (0.48–5.29, p=0.442) | 1.07 (0.27-4.27, p=0.921) | 1 (3.4)* | NE | NE |
| **PFI** | sensitive | 67 (85.9) | - | - | 19 (65.5) |  |  |
|  | resistant | 11 (14.1) | 2.46 (1.26-4.83, **p=0.009**) | 2.92 (1.45-5.91, **p=0.003**) | 10 (34.5) | 3.05 (1.01-9.21, **p=0.048**) | 2.01 (0.59-6.83, p=0.263) |

OS, overall survival; HR, hazard ratio; NLR, neutrophil-to-lymphocyte ratio; PLR, platelet-to-lymphocyte ratio; SII, systemic immune-inflammation index; LIPI, Lung Immune Prognostic Index; PFI, platinum-free interval; NE, not estimable due to complete separation caused by insufficient events in this subgroup. LIPI: poor subgroup results should be interpreted with caution due to very small sample sizes (n=3 for *platinum/pemetrexed and n=1 for *nivolumab/ipilimumab.
